# Impact of malaria and hepatitis B co-infection on clinical and cytokine profiles among pregnant women

**DOI:** 10.1371/journal.pone.0215550

**Published:** 2019-04-19

**Authors:** Nsoh Godwin Anabire, Paul Armah Aryee, Abass Abdul-Karim, Osbourne Quaye, Gordon Akanzuwine Awandare, Gideon Kofi Helegbe

**Affiliations:** 1 West African Centre for Cell Biology of Infectious Pathogens (WACCBIP), University of Ghana, Legon- Accra, Ghana; 2 Department of Biochemistry, Cell & Molecular Biology, University of Ghana, Legon- Accra, Ghana; 3 Department of Biochemistry & Molecular Medicine, School of Medicine and Health Sciences, University for Development Studies, amale- Ghana; 4 Department of Nutritional Sciences, School of Allied Health Sciences, University for Development Studies, Tamale- Ghana; 5 Zonal Public Health Laboratory, Tamale Teaching Hospital, Tamale- Ghana; University of Cincinnati College of Medicine, UNITED STATES

## Abstract

**Background:**

The overlap of malaria and chronic hepatitis B (CHB) is common in endemic regions, however, it is not known if this co-infection could adversely influence clinical and immunological responses. This study investigated these interactions in pregnant women reporting to antenatal clinics in Ghana.

**Methods:**

Clinical parameters (hemoglobin, liver function biomarker, peripheral malaria parasitemia, and hepatitis B viremia) and cytokine profiles were assayed and compared across four categories of pregnant women: un-infected, mono-infected with *Plasmodium falciparum (Malaria group)*, mono-infected with chronic hepatitis B virus (*CHB group*) and co-infected (*Malaria+CHB group*).

**Results:**

Women with *Malaria+CHB* maintained appreciably normal hemoglobin levels (mean±SEM = 10.3±0.3 g/dL). That notwithstanding, Liver function test showed significantly elevated levels of alanine aminotransferase, aspartate aminotransferase and total bilirubin *[P<0*.*001 for all comparisons]*. Similarly, the *Malaria+CHB group* had significantly elevated pro-inflammatory cytokines, including tumour necrosis factor alpha (TNF-α), interleukin (IL)-1β, and IL-6 *[P<0*.*05 for all comparisons]*. In women with *Malaria+CHB*, correlation analysis showed significant negative association of the pro-inflammatory cytokines responses with malaria parasitemia *[IL-1β (P<0*.*001; r = -0*.*645)*, *IL-6 (P = 0*.*046; r = -0*.*394) and IL-12 (P = 0*.*011; r = -0*.*49)]*. On the other hand, the pro-inflammatory cytokine levels positively correlated with HBV viremia *[TNF-α (P = 0*.*004; r = 0*.*549)*, *IL-1β (P<0*.*001; r = 0*.*920)*, *IL-6 (P<0*.*001; r = 0*.*777)*, *IFN-γ (P = 0*.*002; r = 0*.*579)*, *IL-2 (P = 0*.*008; r = 0*.*512) and IL-12 (P<0*.*001; r = 0*.*655)]*. Also, for women in the *Malaria+CHB group*, parasitemia was observed to diminish HBV viremia *[P = 0*.*003*, *r = -0*.*489]*.

**Conclusion:**

Put together the findings suggests that *Malaria+CHB* could exacerbate inflammatory cytokine responses and increase susceptibility to liver injury among pregnant women in endemic settings.

## Background

Coinfections are increasingly being recognized as common risk factors that may contribute to the increased burden of morbidity in pregnancy. In many endemic setting, the overlap of chronic hepatitis virus (HBV) and *P*. *falciparum* infections is common, and an increased prevalence, from 0.7% to 1.7%, of this co-infection has been reported among pregnant women in Ghana [1, 2]. The disease causing pathogens share a common intra-hepatic niche, and each may independently cause liver function test abnormalities [3–9]. Immunologically, both pathogen may also overlap, as each is observed to mainly trigger T helper type 1 (Th1) cytokine responses [10–14]. *P*. *falciparum* causes anemia by reducing red cell counts, while HBV is postulated to increase hemoglobin (Hb) levels by increasing the release of erythropoietin from regenerating hepatic tissues [15–17]. Thus, it may be logical to postulate that HBV could compensate for the effect of *P*. *falciparum* on Hb levels in co-infection state.

Anemia, liver dysfunctions and cytokine imbalance (towards Th1) in pregnancy are associated with significant morbidity and mortality for both the mother and the newborn [18–20]. Though physiologic and biochemical (high serum alkaline phosphatase) changes in pregnancy are often mistaken for signs of liver disease, levels of aminotransferases remain normal, while bilirubin is below normal range [21, 22]. Therefore, elevated levels of aminotransferases and bilirubin in pregnancy gives evidence of liver dysfunction and damage. In normal pregnancy, Th1 and T helper type 2 (Th2) responses are tightly regulated throughout the trimesters [23], but excess of Th1 responses are observed to affect pregnancy outcomes [24].

Although *P*. *falciparum* and HBV are routinely diagnosed in pregnant women on antenatal visits, the hematological and hepatological impact of co-infection has received little attention mainly because: falciparum malaria is curable and does not cause chronic infections, and liver functions tests are not conducted. This study therefore evaluates the influence of co-infection on clinical (Hb levels and liver function) and cytokine profiles in pregnant women. The results of the study provide important information that would be useful in guiding policy/decisions on *P*. *falciparum*/HBV diagnosis, treatment and management in pregnant women in endemic countries.

## Methods

### Study sites and participants

Pregnant women were recruited on their first antenatal visit at various hospitals and health centres in the Northern Region of Ghana, from October 2016 to February 2017. The study areas were Tamale metropolis and Central Gonja District. Recruitments were done at Tamale Teaching Hospital, Tamale Central Hospital, Tamale West Hospital and Bilpella Health Centre, all located in Tamale metropolis. In the Central Gonja District, the participants were recruited from Sankpala and Kosawgu Health Centres. A total of 2071 pregnant women were screened for *P*. *falciparum* and HBV infections. Following the exclusion of women with documented chronic alcoholism, diagnosed eclampsia/preeclampsia, chronic degenerative diseases, use of hepatotoxic or immunosuppressant drugs, sickle cell trait, and other viral (HIV, hepatitis C) and parasitic (amebiasis, hydatid cyst, ascaris and schistosomiasis) infections, 257 were enrolled and used for this study. Also, demographic and obstetric data were obtained from the participants’ antenatal care log books.

### Ethical consideration

Informed consent was obtained from each study participant after an explanation of the purpose, benefits, and risks of the study was provided. The ethics committee of the Tamale Teaching Hospital approved the study (Approval ID: TTHERC/21/04/16/02).

### Clinical and DNA samples

Stool and urine samples were collected and screened for the other parasitic infections that are listed in the exclusion criteria. Venous blood samples (5 mL) were collected and a portion (4 mL) was processed for serum. The serum was used for the diagnosis of CHB, liver function test and quantification of cytokines. The remaining blood (1 mL) was used for diagnoses of malaria, and screening of sickle cell trait and the other viral infections captured in the exclusion criteria. DNA was extracted, from both whole blood and serum, with the QIAamp DNA mini kit (Qiagen, Hilden, Germany) according to the manufacturer’s protocol. The extracted DNA samples were used in the PCR assays for plasmodium species detection and HBV quantification.

### Clinical laboratory analyses

CareStart histidine-rich protein 2 (HRP-2) test cassettes (Access Bio Inc., New Jersey, USA) and hepatitis B surface antigen (HbsAg) test strips (Ark Biotech., Shanghai, China) were used for rapid diagnoses of malaria and hepatitis B respectively. Both tests were performed in accordance with the manufacturer’s recommendations.

Malaria parasite densities were determined by microscopy, performed by two independent World Health Organization (WHO) certified microscopists based on WBC count of 8,000 cells/μL [25].

Hemoglobin was quantified by the cyanomethemoglobin (Drabkin’s) method, using BS-3000 Chemistry Analyzer (Sinnowa Medical Science and Technology Co. Ltd, Nanjing, China). Five (5) uL of EDTA blood and standards were thoroughly mixed in separate plain tubes containing 5 ml Drabkin’s solution (Sigma-Aldrich, St. Louis, USA) and allowed to incubate at room temperature for 30 minutes. Absorbances were read at 540 nm and the hemoglobin concentrations were obtained using a calibration curve. Samples were measured in duplicates, and those with standard deviations <15% were used in the data analysis.

Serum levels of alanine aminotransferase (ALT), aspartate aminotransferase (AST), alkaline phosphatase (ALP) and total bilirubin (Tbil) were measured using the Selectra Pro X5 automated chemistry analyzer (Elitech Group of Companies, Puteaux, France). The instrument was calibrated and standardized daily before the measurements were obtained. Samples were quantified in duplicates and duplicate samples with <15% percentage coefficient of variation were considered.

### PCR for plasmodium species detection

Plasmodium species were detected using a previously described nested PCR method [26, 27]. In the first PCR, the small sub-unit ribosomal genes of plasmodium were amplified using genus-specific forward and reverse primers. In the second PCR, species-specific forward and reverse flanking primers were used for detection of *P*. *falciparum*, *P*. *vivax*, *P*. *malariae*, and *P*. *ovale*. For both rounds of PCR, the total reaction volume was 20 μL, containing 2.5 μL of 10X PCR buffer, 1.5 μL of 25 mM MgCl_2_, 0.5 μL of 10 μM primers, 0.5 μL of 10 μM deoxynucleotides and 0.2 μL of 1 U Taq DNA polymerase (Qiagen, Hilden, Germany). The PCR products were visualized on 2% agarose gel containing 1.5 μL ethidium bromide, and the plasmodium species were identified by respective band sizes.

### PCR for quantification of HBV DNA

A PCR assay that was described previously was used to amplify a 98 base pair product of the S-gene of HBV genome [28]. The total reaction volume of 10 μL consisted of 0.5 μL each of 10 μM forward and reverse primers, 5 μL Low ROX PerfeCTa syber green SuperMix (Quanta Biosciences Inc., Gaithersburg, USA), 2 μL nuclease free water and 2 μL DNA. Thermal cycling was performed on QuanStudio 5 TaqMan quantitative real-time PCR system (Thermofisher Scientific, New Jersey, USA). Cycling conditions were: initial denaturation at 95°C for 15 minutes, followed by 40 cycles of denaturation at 94°C for 15 seconds, annealing at 55°C for 30 seconds, and elongation at 68°C for 30 seconds. The AcroMetrix HBV High Control (Thermofisher Scientific, New Jersey, USA) with viral load of 7.43 log _(10)_ IU/mL was serially diluted with nuclease free water to four different dilutions of 1/5, 1/25, 1/125, and 1/625 with final concentrations of 6.82, 6.17, 5.52 and 4.87 log _(10)_ IU/mL, respectively. Before assaying the samples, the study assessed the performance of the assay by comparing it with the Roche Molecular Systems for detection of HBV DNA. The co-efficient of variation between both assays was <5%.

### Levels of human anti-HBV virus core antibody (HBcAb)

Sera of pregnant women who tested positive for HBV infection by PCR were each tested for the presence of HBcAb-IgG and HBcAb-IgM using IgG-HBcAb ELISA kit and IgM- HBcAb ELISA kit (MyBioSource Inc., San Diego, USA), respectively, following the manufacturer’s protocols. Absorbances were read at 450 nm using a Varioscan lux plate reader (Thermofisher Scientific, New Jersey, USA). Each sample was tested in duplicate, and those with standard deviations <15% were considered. The results obtained were interpreted in accordance with the manufacturer’s recommendations, and all samples tested were IgG positive and IgM negative.

### Cytokine analysis

Serum samples were thawed and clarified by centrifugation at 14, 000 rpm for 10 minutes and used to determine the levels of 10 cytokines; interferon gamma (IFN-γ), Tumor necrosis factor alpha (TNF-α), interleukin (IL)-1β, IL-6, IL-12, IL-2, IL-10, IL-4, IL-5 and IL-13. The cytokines were measured on the xMAP Technology platform (Luminex Corporation, Austin, USA) using the magnetic beaded Milliplex MAP 13-plex kit (Merck, Darmstadt, Germany), and following the manufacturer’s protocol. Twenty-six samples of each category of the study participants were randomly selected for the assay. The samples were assayed in duplicates and those with <15% percentage coefficient of variation were included in the data analysis.

### Statistical analysis

Categorical variables were presented as frequencies and percentages, and compared by Pearson’s chi-square test. Continuous parametric data were described as mean and standard deviation (SD) or standard error of mean (SEM), and compared by one-way analysis of variance (ANOVA) or Tukey's multiple comparisons test with Tukey's correction. Continuous non-parametric data were presented as median and interquartile range (IQR), and compared by Dunn's multiple comparison tests with Dunn’s correction. Correlation analyses were done by Spearman’s test.

## Results

### Infection statuses and obstetric information

Of the 257 women enrolled, 73 were uninfected, 80 were mono-infected with *P*. *falciparum* (*Malaria group*), 68 were mono-infected with HBV (*CHB group*), and 36 were co-infected with both pathogens (*Malaria+CHB group*). The infection statuses of the pregnant women were independent of age, gestation and gravidity (Table 1). All the women in the *CHB* and *Malaria+CHB groups* were IgG positive and IgM negative, which suggests that they had chronic HBV infections.

**Table 1:** Distribution of demographic and obstetric characteristics in the different groups of pregnant women.

Parameters	un-infected (*n* = 73)	*Malaria group* (*n* = 80)	*CHB group* (*n* = 68)	*Malaria+CHB group* (*n = 36*)	^α/β^P
Age (years), mean ± SD	27.6 ± 4.9	25.5 ± 5.3	26.7 ± 5.1	26.7 ± 5.7	^α^0.103
Gravidity, n (%)					
Primigravida	12 (16.4%)	26 (32.5%)	16 (23.5%)	9 (25.0%)	^β^0.147
Multigravida	61 (83.6%)	54 (67.5%)	52 (76.5%)	27 (75.0%)	
Gestation, n (%)					
First trimester	26 (35.6%)	24 (30.0%)	31 (45.6%)	12 (33.3%)	^β^0.257
Second trimester	34 (46.6%)	46 (57.5%)	32 (47.1%)	21 (58.3%)	
Third trimester	13 (17.8%)	10 (12.5%)	5 (7.4%)	3 (8.3%)	

α: analyzed by one-way ANOVA

β: analyzed by Pearson’s chi-square or Fisher’s exact test

SD: standard deviation

n = number of samples

Statistical significance was considered at P <0.05.

### Hemoglobin levels

**Fig 1:**
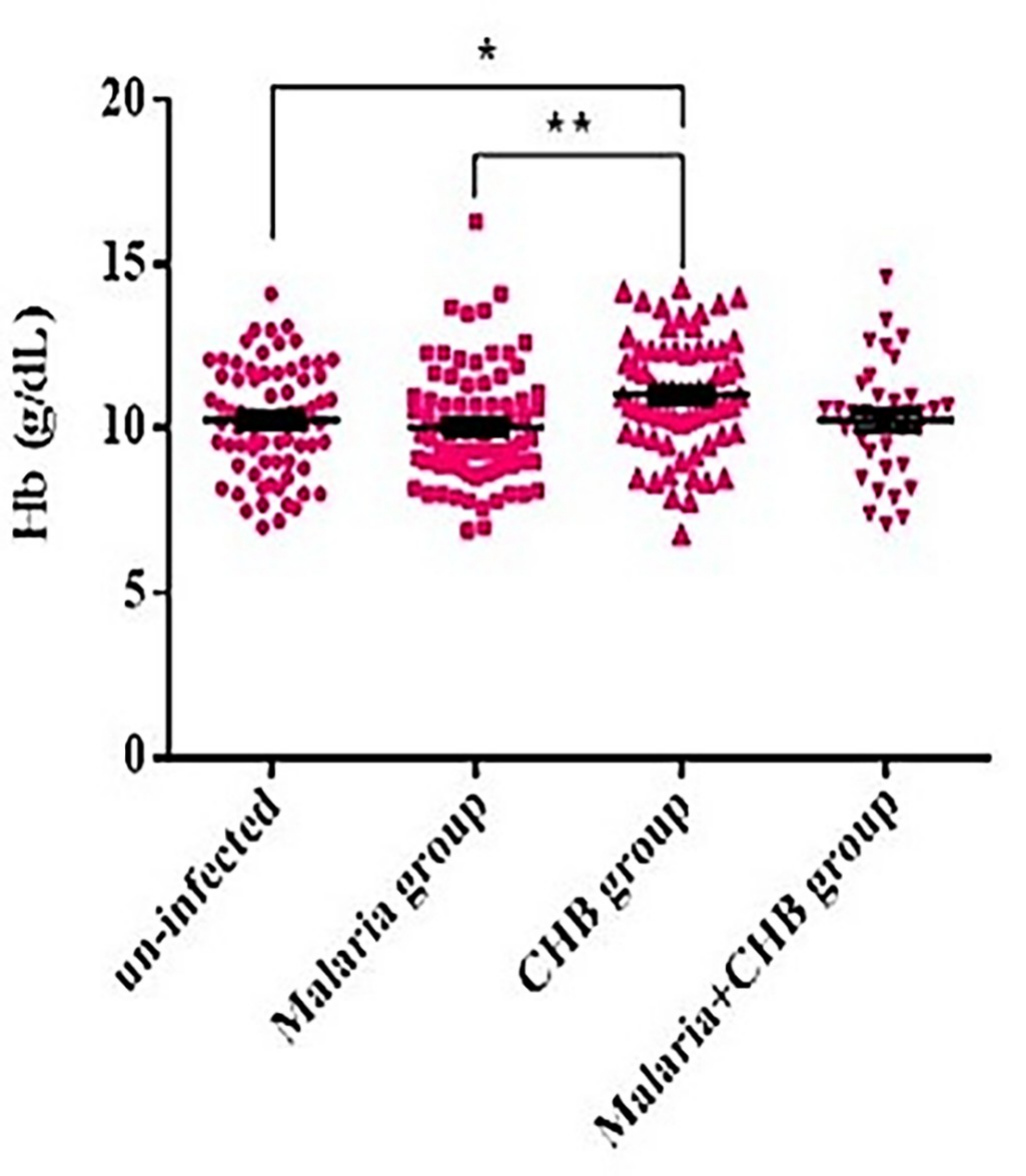
Comparison of levels of hemoglobin (Hb) amongst the groups of pregnant women. Pair-wise differences between the groups was tested by the Tukey's multiple comparisons test with Tukey's correction, *P = 0.043, **P = 0.004. Horizontal bar with error bars represents the mean and standard error of mean respectively.

The mean Hb ± SEM was significantly higher in the *CHB group* (11.0 ± 0.2 g/dL) than the un-infected (10.2 ± 0.2 g/dL) and the *Malaria group* (10.0 ± 0.2 g/dL) (Fig 1). However, Hb levels were statistically similar between the *CHB* and *Malaria+CHB groups* (10.3 ± 0.3 g/dL), *P = 0*.*129*. In addition, a higher proportion of women with *CHB* had normal Hb levels (Hb **≥** 11g/dL) (χ^2^  =  12.47, *P***<** 0.006, S1 Table).

### Serum levels of liver and inflammatory biomarkers

**Fig 2:**
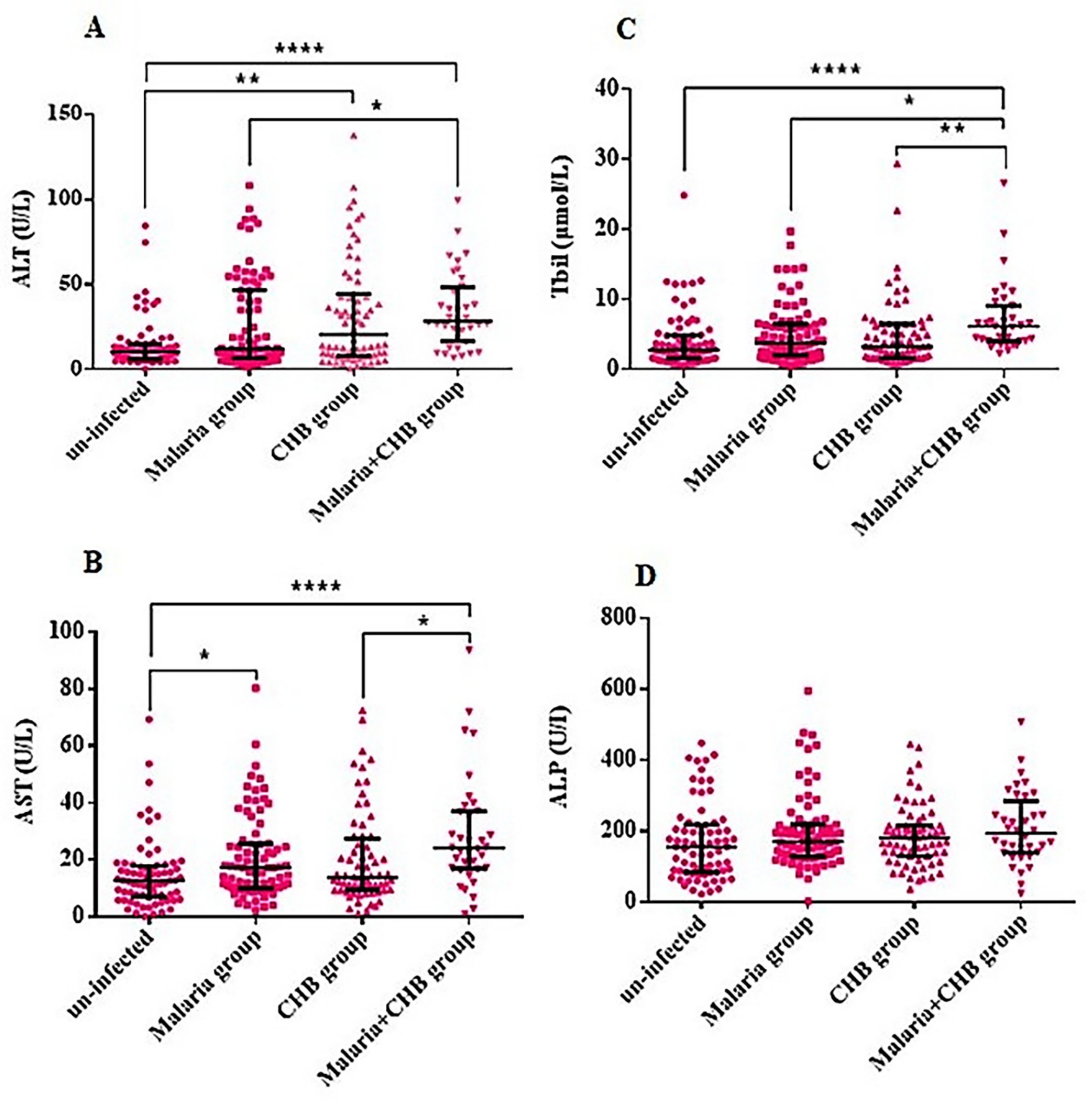
Differences in levels of liver biochemical parameters across the different categories of pregnant women. Dunn's multiple comparison tests for pair-wise differences in serum levels of (A) alanine aminotransferase (ALT), (B) aspartate amino transferase (AST), (C) total bilirubin (Tbil) and (D) alkaline phosphatase (ALP). Significant P-values were observed at *P < 0.05, **P < 0.01 and ****P < 0.0001. Horizontal bar with error bars represents the median and the interquartile range respectively.

**Fig 3:**
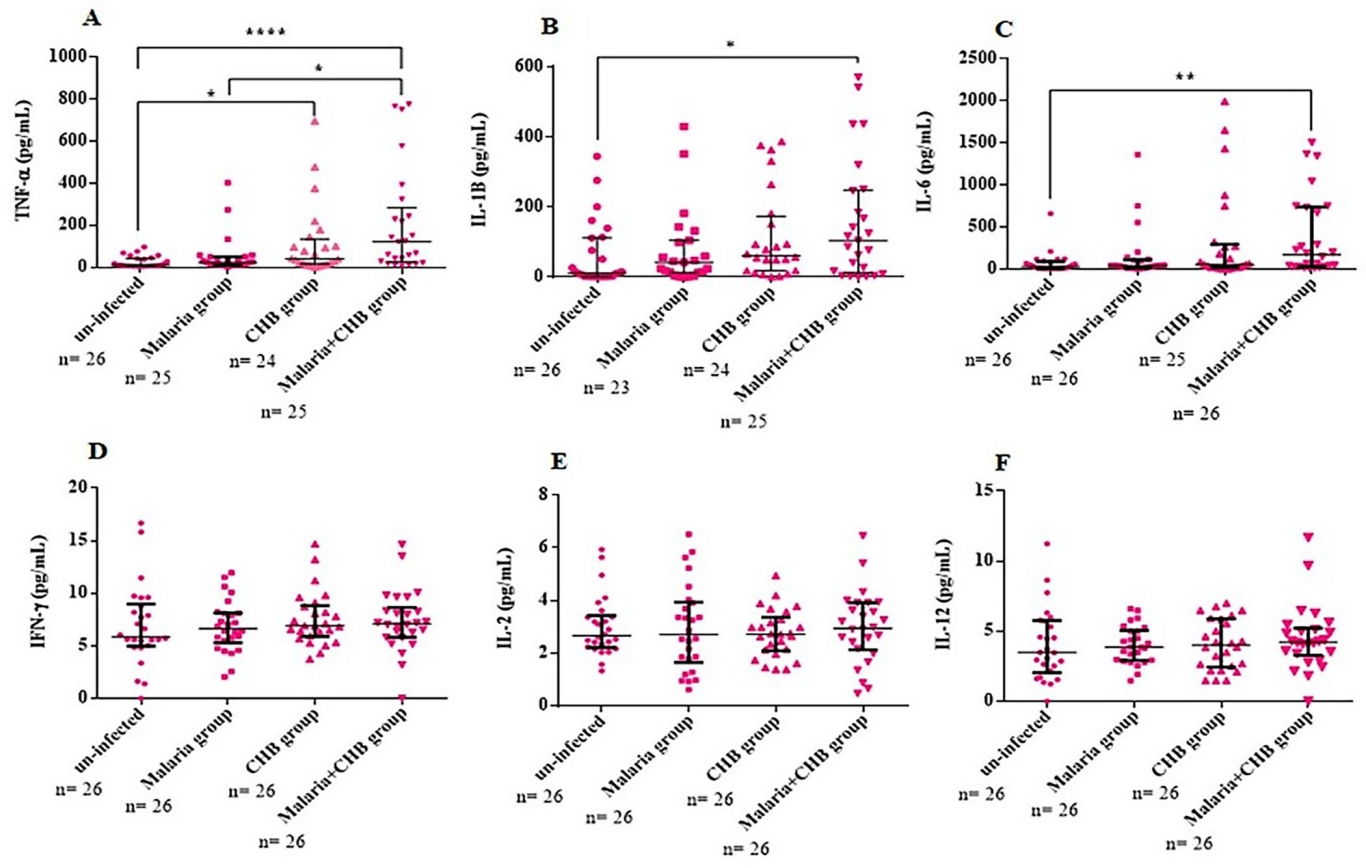
Differences in levels of pro-inflammatory cytokines amongst the groups of pregnant women. Dunn's multiple comparison tests for differences in serum levels of (A) tumour necrosis factor alpha (TNF-α), (B) interleukin (IL)-1β, (C) IL-6, (D) interferon gamma (IFN-γ), (E) IL-2 and (F) IL-12. Statistical significance was observed at *P< 0.05, **P< 0.01 and **** P< 0.0001. Horizontal bar with error bars represents the median and the interquartile range respectively. n = number of samples of each category of the study participants assayed.

**Fig 4:**
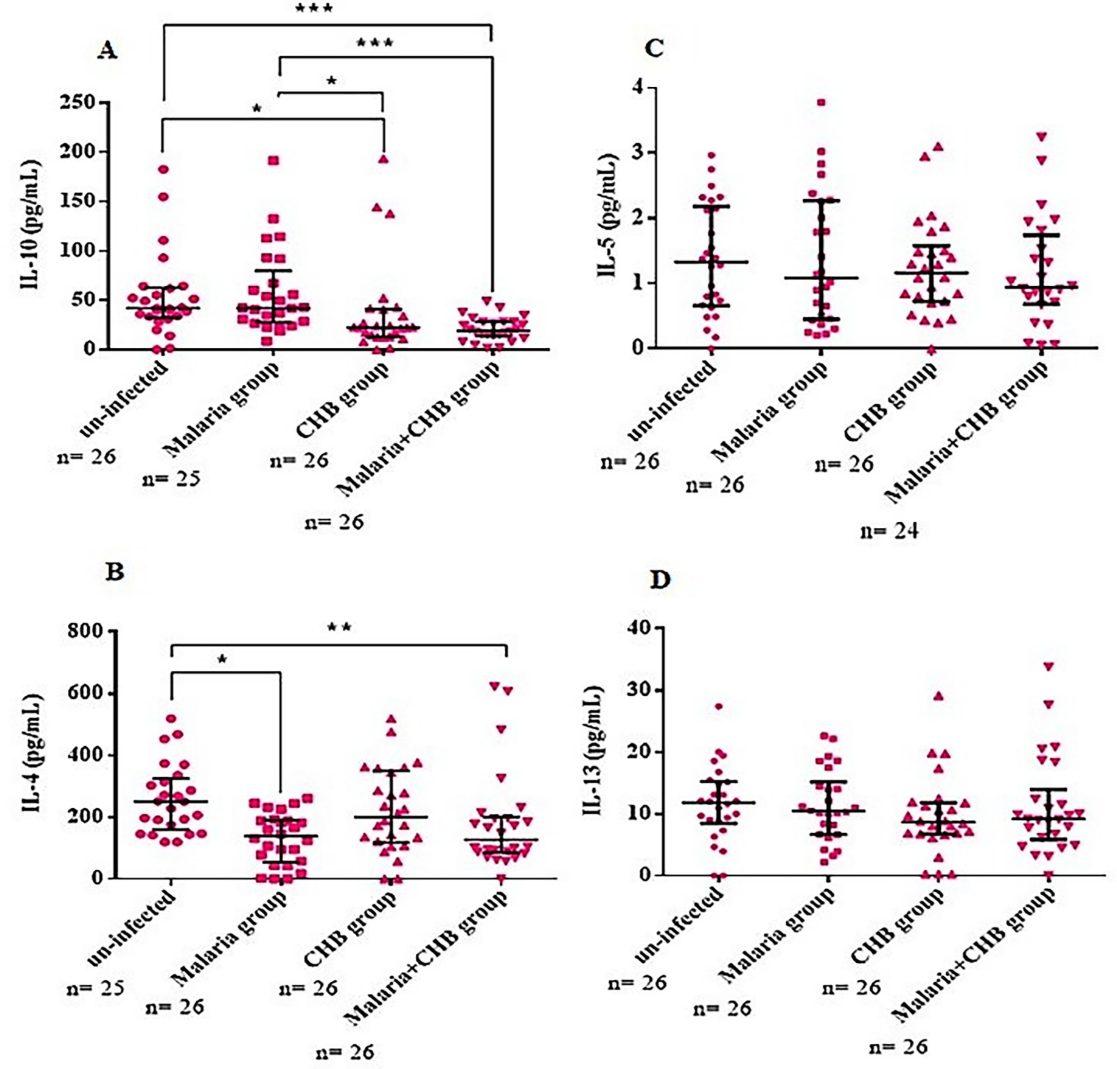
Comparison of serum levels of anti-inflammatory cytokines in the different categories of pregnant women. Dunn's multiple comparison tests for significant P-values in levels of (A) IL-10, (B) IL-4 (C) IL-5 and (D) IL-13. Statistical significance was observed at *P < 0.05, ** P < 0.01 and ***P < 0.001. Horizontal bar with error bars represents the median and the interquartile range respectively. n = number of samples of each category of the study participants assayed.

Compared with the other groups, the *Malaria+CHB group* had significantly elevated levels of the liver biochemical parameters including ALT (Fig 2A), AST (Fig 2B) and Tbil (Fig 2C). Similarly, the group had elevated serum levels of pro-inflammatory cytokines including TNF-α (Fig 3A), IL-1β (Fig 3B), and IL-6 (Fig 3C). However, compared with the other groups, anti-inflammatory cytokines including IL-10 (Fig 4A) and IL-4 (Fig 4B), were lower in women with *Malaria+CHB*. Women with *Malaria* and *CHB* had similar levels of the liver biomarkers and cytokines, except for significantly higher levels of IL-10 in the *Malaria group* (Fig 4A).

### Correlation of pro-inflammatory cytokine responses with malaria or HBV intensity in co-infected cohorts

In women with *Malaria+CHB*, Levels of 3 out of the 6 pro-inflammatory cytokines negatively correlated with malaria parasitemia [IL-1β (*P<0*.*001*; *r = -0*.*645)*, IL-6 (*P = 0*.*046; r = -0*.*394)* and IL-12 (*P = 0*.*011; r = -0*.*49*)]. That notwithstanding, parasitemia was similar between the *Malaria+CHB* and *CHB groups (P = 0.304, Table 2)*. On the other hand, levels of all the pro-inflammatory cytokines positively correlated with HBV viremia [TNF-α *(P = 0*.*004; r = 0*.*549)*, IL-1β *(P<0*.*001; r = 0*.*920)*, IL-6 *(P<0*.*001; r = 0*.*777)*, IFN-γ *(P = 0*.*002; r = 0*.*579)*, IL-2 *(P = 0*.*008; r = 0*.*512)* and IL-12 (*P<0*.*001; r = 0*.*655)*], and viremia was significantly lower in the *Malaria+CHB* compared to the *CHB group* (*P = 0*.*016*, Table 2). Further analysis showed that malaria parasitemia negatively correlated with hepatitis B viremia in women with *Malaria+CHB* (*P = 0*.*003*, r = -0.489).

**Table 2:** Differences in malaria parasitemia and hepatitis B viremia amongst the different categories of pregnant women.

Parameters	*Malaria group* (*n* = 80)	*CHB group* (*n* = 68)	*Malaria+CHB group* (*n = 36*)	P
Malaria parasite count (parasites/μL), Median (IQR)	1310 (490–2560)		860 (420–1870)	0.304
Hepatitis B viremia [Log_10_ mean (IU/mL)], Median (IQR)		4.65 (4.09–5.24)	4.05 (3.54–5.09)	0.016

P: analyzed by Mann Whitney test, considered statistically significant at <0.05

IQR: interquartile range.

n = number of samples

## Discussion

Maternal anemia is an issue of important public health relevance. During pregnancy, women with Hb <11g/dL are diagnosed as anemic while those with Hb **≥** 11g/dL are considered to have normal level [29]. *P*. *falciparum* causes anemia by reducing red cell counts, HBV on the other hand is postulated to increase Hb levels by increasing the release of erythropoietin from regenerating hepatic tissues [15–17]. It is therefore logical that pregnant women with *Malaria+CHB* maintained intermediate levels of hemoglobin (above the *Malaria group* and un-infected, and below *CHB group*). This observation may suggest that in women with *Malaria+CHB*, activities of the plasmodium parasites in reducing red cell count may be compensated for by the activities of the virus, thus maintaining an appreciable hemoglobin concentration. The significant association of *CHB* with Hb ≥11g/dL further justifies the possible impact of HBV in increasing hemoglobin levels among the pregnant women. However, this might not necessarily translate into improved pregnancy outcomes, since cases of gestational diabetes, antepartum hemorrhage and preterm delivery are observed to be more frequent in pregnant women with *CHB* [30–32].

Relative to the un-infected (Figs 2 and 3), the increased serum levels of the liver function biomarkers and pro-inflammatory cytokines in the *Malaria* or *CHB groups* have been previously reported [3–6, 8–14]. In particular, elevated levels of IL-10 among the *Malaria group* is a common finding amongst African multigravidae mothers with *P*. *falciparum* infection [14, 33–35], and as such, the cytokine has been implicated in the immunopathology of placental malaria [36].

Similarity in levels of the liver biomarkers between women with *Malaria* and *CHB* may lend credence that the elevated levels in those with *Malaria+CHB* resulted from additive activities of both pathogens. The synergistic elevation in levels of the liver biomarker in the *Malaria+CHB* group may be attributed to immune factors that limit the infections at the liver-stage. Particularly, natural killer (NK) and natural killer T (NKT) cells which are abundantly available in the liver, interact with pathogens and initiate liver-stage cell-mediated immunity [37, 38]. For HBV infections, NK cells contribute to liver inflammation by tumor necrosis factor–related apoptosis-inducing ligand (TRAIL)-mediated death of hepatocytes, which is non-antigen–specific, and can be switched on by a milieu of cytokines [39]. Therefore, it is possible that, in women with *Malaria+CHB*, cytokines released in response to *P*. *falciparum* could further activate the apoptosis of HBV-infected hepatocytes, and exacerbate liver damage.

While peripheral levels of TNF-α, IL-1β and IL-6 were similar in women with *Malaria* and *CHB*, the pro-inflammatory cytokines were significantly increased in those with *Malaria+CHB*, which further suggests a possible additive effect of the infections. The correlation analysis with the *Malaria+CHB group* suggests that increased pro-inflammatory cytokine levels as a necessary immune response against malaria helped in reducing HBV intensity. This corroborates with studies indicating that *P*. *falciparum* malaria modulates viremia in chronic hepatitis B virus infection [40, 41]. IL-10 and IL-4 are key anti-inflammatory cytokines that regulate the activities of pro-inflammatory cytokines responses. Thus, the diminished peripheral levels of IL-10 and IL-4 in the women with *Malaria+CHB* may suggest susceptibility to cytokine imbalance (towards Th1). In particular, placental cytokine imbalance is noted to be associated with threatened abortion, recurrent spontaneous miscarriage, and preterm delivery [18, 42]. That notwithstanding, biased peripheral pro-inflammatory cytokine responses has been implicated with pregnancy complications [43, 44].

## Limitations

A longitudinal approach on changes of Hb levels in the co-infected cohorts after clearance of *P*. *falciparum* would have been paramount in substantiating the compensatory effect of the viral infection. Again such an approach would have enabled us to see the kinetics of *Malaria+CHB* co-infection and to identify times when the markers of liver injury and inflammatory responses are enhanced. In addition, such an approach would have enabled us to substantiate our findings by profiling placental cytokines responses, malaria parasitemia and HBV viremia, and associating the results with pregnancy complications. Also profiling of innate immune cells and T-cell populations would have given a better insight of the effect of the co-infection on the immune response. Nonetheless, the current study provides evidence on the need to take into cognizance the possible deleterious impact of *Malaria+CHB* on pregnant women. In this regard, liver functions test which is important in guiding diagnosis and treatment of liver diseases in pregnancy must be readily accessible to pregnant women on ANC clinics.

## Conclusion

Put together the findings suggests that *Malaria+CHB* could exacerbate inflammatory cytokine responses and increase susceptibility to liver injury among pregnant women in endemic settings.

## Supporting information

S1 Table Association between infection type and normal hemoglobin levels during pregnancy. (DOCX)
