# Supplementary material for: Impact of malaria and hepatitis B co-infection on clinical and cytokine profiles among pregnant women
Source: PLoS One. 2019 Apr 19;14(4):e0215550. doi: 10.1371/journal.pone.0215550 (PMC6474591; doi:10.1371/journal.pone.0215550)
Supplement: S1 Table — (DOCX) [file pone.0215550.s001.docx]

**S1 Table. Association between infection type and normal hemoglobin levels during pregnancy.**

| Infection type | Hb ≥11g/dL | | χ^2^ | P |
| --- | --- | --- | --- | --- |
|  | Yes, n (%) | No, n (%) |  |  |
| *CHB group* | 35 (51.5) | 33 (48.5) | 12.47 | 0.006 |
| *Malaria group* | 20 (25.0) | 60 (75.0) |  |  |
| *Malaria+CHB group* | 10 (27.8) | 26 (72.2) |  |  |
| Un-infected | 25 (34.2) | 48 (65.8) |  |  |

P: analyzed by Pearson Chi square test, and considered significant at < 0.05.

**χ^2^:** Chi square statistic
